# Acute presentation of a heterotopic pregnancy following spontaneous conception: a case report

**DOI:** 10.1186/1757-1626-2-9369

**Published:** 2009-12-21

**Authors:** Sameer Umranikar, Aarti Umranikar, Junaid Rafi, Pauline Bawden, Shalini Umranikar, Ben O'Sullivan, Adam Moors

**Affiliations:** 1 Department of Obstetrics & Gynaecology, Princess Anne Hospital, Coxford Road Southampton SO16 5YA, UK; 2 Department of Obstetrics & Gynaecology, North Hampshire and Basingstoke Foundation Trust Hospital, Aldermaston Road Basingstoke RG24 9NA, UK

## Abstract

Spontaneous heterotopic pregnancy is a rare clinical condition in which intrauterine and extra uterine pregnancies occur at the same time. It can be a life threatening condition and can be easily missed with the diagnosis being overlooked. We present the case of a 40 year old patient who was treated for a heterotopic pregnancy. She had a transvaginal ultrasound because of a previous ectopic pregnancy and an intrauterine gestational sac was seen with false reassurances. The patient presented acutely with a ruptured tubal pregnancy and this was managed laparoscopically. The ectopic pregnancy was not suspected at her initial presentation. A high index of suspicion is needed in women with risk factors for an ectopic pregnancy and in low risk women who have free fluid with or without an adnexal mass with an intrauterine gestation.

## Case presentation

A 40-year-old Caucasian lady of UK origin was seen in our Early Pregnancy Unit at 8 weeks gestation to confirm site of pregnancy as she had a previous ectopic pregnancy. This was a spontaneous conception with no previous fertility treatment. She did not use any contraception in the interim.

Three years earlier she was diagnosed with a right tubal ectopic pregnancy which was treated by a laparoscopic salpingectomy. Prior to that she had two normal pregnancies and deliveries. She did not have any risk factors for an ectopic pregnancy at that time. The left fallopian tube looked normal at the time of the laparoscopy.

**Figure 1:**
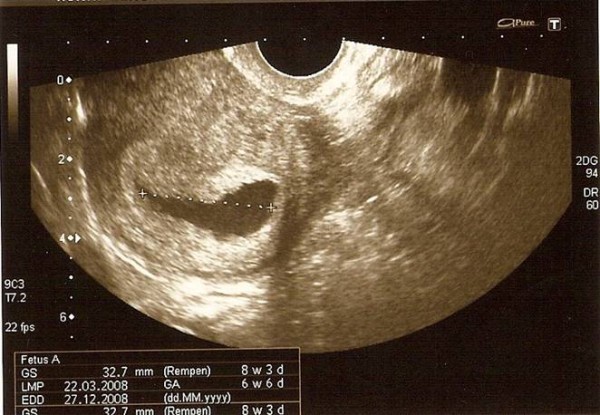
**Transvaginal ultrasound of uterus showing an irregular intrauterine gestational sac of approximately 8 weeks**.

She was completely asymptomatic at the initial consultation. A transvaginal scan showed an irregular gestational sac of approximately 8 weeks with a yolk sac but no fetal pole (Figure 1). There was a corpus luteal cyst noted in the left ovary with a small amount of free fluid in the Pouch of Douglas. The right ovary was normal with no obvious adnexal masses seen. A rescan was scheduled in 10 days time to check for viability of the pregnancy.

However six days later the patient presented as an emergency with acute left sided abdomino-pelvic pain and generally feeling unwell. She had no vaginal bleeding. On examination she was cold, clammy and hypotensive. Abdominal examination was suggestive of an acute abdomen with severe tenderness, guarding and rigidity. Clinical differential diagnosis at that stage was a possible bleed from the corpus luteal cyst which was seen in the initial scan or an ectopic pregnancy.

**Figure 2:**
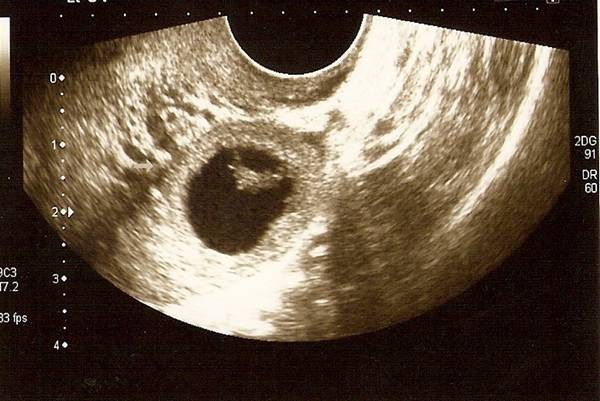
**Transvaginal ultrasound showing left adnexal mass containing gestational sac with fetal pole**.

A transvaginal scan done by the same senior sonographer showed the intrauterine gestational sac similar to the previous scan few days earlier. In addition however there was a left adnexal mass with a gestational sac and a fetal pole suggestive of an ectopic pregnancy (Figure 2). There was a faint fetal heart seen in the fetal pole. The amount of free fluid in the pelvis was significantly more as compared to her previous scan.

She was counseled and consented for an operative laparoscopy and ERPC (evacuation of retained products of conception).

**Figure 3:**
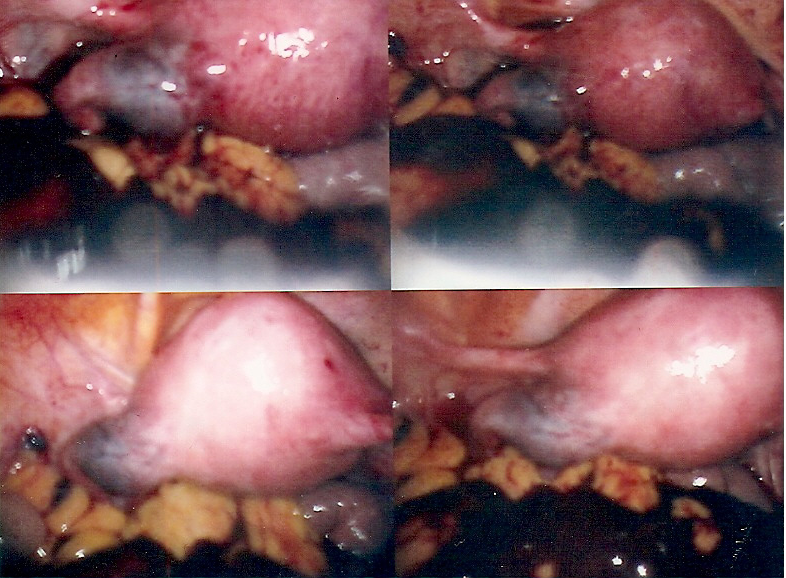
**Laparoscopic findings of left tubal ectopic pregnancy in the isthmic area of the left fallopian tube with hemoperitoneum**.

At laparoscopy (Figure 3) there was approximately a litre of hemoperitoneum. There was a 3-4 cm left tubal isthmic ectopic pregnancy seen. Both the ovaries appeared normal with an absent right fallopian tube.

**Figure 4:**
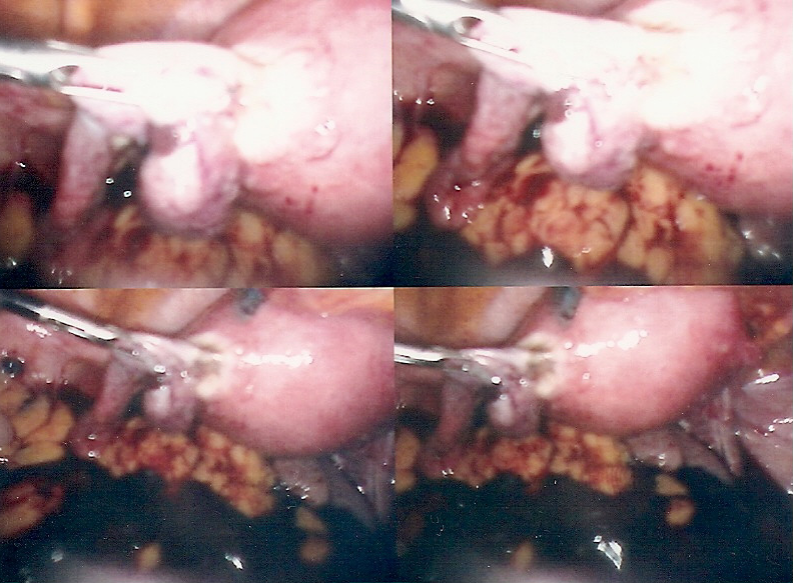
**Laparoscopic left salpingectomy being undertaken**.

A total left salpingectomy (Figure 4) was performed laparoscopically followed by and ERPC. A serum beta hCG taken just before surgery was 21846 iu.

The patient made an unremarkable recovery from the surgery and was discharged the following day. Both the tissue specimens were sent separately and the histology from each of the specimens confirmed chorionic villi suggestive of a heterotopic pregnancy.

## Discussion

Heterotopic pregnancy is defined as the presence of multiple gestations, with one being in the uterine cavity and the other outside the uterus commonly in the fallopian tube and uncommonly in the cervix or ovary [1-3]. Spontaneous triplet heterotopic pregnancy has also been reported with two yolk sacs seen in one tube [4], and in another case an ectopic pregnancy in each tube with a single intrauterine gestation [5].

Heterotopic pregnancies are becoming more common following assisted conception techniques for subfertility [6], however spontaneous heterotopic pregnancies are quite rare [7]. The incidence quoted is 1 in 30,000 pregnancies [8].

Heterotopic pregnancies can pose a diagnostic dilemma because an early transvaginal ultrasound may not diagnose an ex-utero gestation in all cases. A diagnosis of a pseudosac should be made with caution, as even in the presence of a pseudo sac there can be a high false positive diagnosis of an ectopic pregnancy [9]. Sometimes the presence of a haemorrhagic corpus luteum can confuse and delay the diagnosis of a heterotopic pregnancy [10].

The detection rate of heterotopic pregnancy can vary from 41 to 84% with transvaginal ultrasound scans [12,13]. It is influenced by factors like routine and easy access to transvaginal ultrasound scans for high risk patients with a history of previous ectopic pregnancy and those who received fertility treatment.

With an increase in assisted conception the likelihood of detecting heterotopic pregnancy will increase but missed or delayed diagnosis of spontaneous heterotopic pregnancy remains a diagnostic dilemma and a challenge for gynaecologists. In a case series Louis-Sylvestre et al [11], mentioned 13 cases of heterotopic pregnancy out of which only one case was a spontaneous heterotopic pregnancy, 6 with ovulation induction and 6 with IVF. The mean gestational age at the time of the diagnosis was 8 weeks and 54% heterotopic pregnancies were detected by transvaginal ultrasound. All the patients underwent surgical treatment out of which 10 had a laparoscopy and 3 had a laparotomy mainly for significant hemoperitoneum. They found laparoscopy to be useful for the early diagnosis of heterotopic pregnancy and resulted in good surgical outcomes.

The question however arises in women with spontaneous gestations who do not necessarily have early ultrasound scans. Women with previous ectopic pregnancy, tubal surgery or previous pelvic inflammatory disease may be at a higher risk and should be scanned at an early gestation to confirm the location of the pregnancy. Also a high index of suspicion is necessary in the low risk symptomatic patient with abdominal or pelvic pain in which ultrasound findings are consistent with intrauterine gestation sac while free fluid is noted in the pelvis with or without an adnexal mass.

The diagnostic role of serum beta hCG levels in heterotopic pregnancy is debatable [14]. The normal algorithm for the rapid rise in the serum beta hCG in early pregnancy cannot be used due to the presence of the intrauterine gestation which could lead to false assurances. This can be highlighted in our case where the serum beta hCG was noted to be 21846 IU/l just before the laparoscopy was undertaken. The important learning point from our case was that the diagnosis was not suspected at the initial presentation and the patient presented subsequently with acute abdominal pain with intra peritoneal haemorrhage. The finding of an intrauterine gestational sac was a red herring and led to false assurances. In women with a previous ectopic gestation treated surgically or non surgically, increased vigilance is required even if they are asymptomatic and an intrauterine gestation is confirmed. Similarly if a patient continues to have ongoing abdominal or pelvic pain with a confirmed intrauterine pregnancy, one of the differential diagnoses should be heterotopic pregnancy.

## Competing interests

The authors declare that they have no competing interests.

## Authors' contributions

SaU and ShU were involved in the treatment of the patient and with AU and JR contributed in writing the manuscript. PB helped to research the articles. BS and AM contributed towards the technical guidance for the case report. All authors read and approved the final manuscript.

## Consent

Written informed consent was obtained from the patient for publication of this case report and accompanying images. A copy of the written consent is available for review from the journal's Editor-in-Chief.
